# Sex, Sport, IGF-1 and the Community Effect in Height Hypothesis

**DOI:** 10.3390/ijerph120504816

**Published:** 2015-05-04

**Authors:** Barry Bogin, Michael Hermanussen, Werner F. Blum, Christian Aßmann

**Affiliations:** 1 Centre for Global Health & Human Development, School of Sport, Exercise & Health Sciences, Loughborough University, Loughborough LE11 3TU, UK; 2 Department of General Pediatrics, Christian-Albrechts-Universität Kiel, Kiel 24105, Germany; E-Mail: michael.hermanussen@gmail.com; 3 Center of Child and Adolescent Medicine, Justus-Liebig-University, Giessen 61250, Germany; E-Mail: wernblum@gmail.com; 4 Department of Statistics and Econometrics, Otto-Friedrich-Universität Bamberg, Bamberg 96045, Germany; E-Mail: christian.assmann@uni-bamberg.de

**Keywords:** GH/IGF-1, social dominance, social networks

## Abstract

We test the hypothesis that differences in social status between groups of people within a population may induce variation in insulin-like growth factor-1(IGF-1) levels and, by extension, growth in height. This is called the community effect in height hypothesis. The relationship between IGF-1, assessed via finger-prick dried blood spot, and elite level sport competition outcomes were analysed for a sample of 116 undergraduate men and women. There was a statistically significant difference between winners and losers of a competition. Winners, as a group, had higher average pre-game and post-game IGF-1 levels than losers. We proposed this type of difference as a proxy for social dominance. We found no evidence that winners increased in IGF-1 levels over losers or that members of the same team were more similar in IGF-1 levels than they were to players from other teams. These findings provide limited support toward the community effect in height hypothesis. The findings are discussed in relation to the action of the growth hormone/IGF-1 axis as a transducer of multiple bio-social influences into a coherent signal which allows the growing human to adjust and adapt to local ecological conditions.

## 1. Introduction

Human growth in height, weight and other body dimensions are widely used as indicators of well-being in environmental epidemiology and public health research [1,2,3,4,5]. Social, economic and political differences between human groups are often associated with differences in the mean heights of these groups [6,7,8,9]. This is why James Tanner described human growth in height, ‘as a mirror of the condition of society’ [10,11].

Growth hormone (GH) and especially its primary mediator insulin-like growth factor-1 (IGF-1) have fundamental roles in the regulation of metabolism and growth of the human body [12,13,14]. Prior to adulthood, a deficiency of GH, IGF-1, their cell receptors, or their signal transducers (JAK2, STAT5b, *etc.*) results in growth retardation and short stature. Children and adolescents with pituitary gigantism have an excessive production of GH, and IGF-1 levels are elevated. Clinical studies demonstrate the central role of GH and IGF-1 in human growth. Peripubertal children with idiopathic short stature treated with GH show significant increases in adult height in randomized, double-blind, placebo-controlled trials [15]. In a randomized, controlled, multicentre clinical trial [16,17], children with short-stature, but not deficient in GH were treated with GH or not treated. Over a 5 year period, the GH recipients showed significant increase in height in a dose-response fashion. The change in IGF-1 levels from baseline explained the largest amount of the variance (28% of the total variance) in greater height compared with untreated children. The authors of this study interpret the findings to indicate that GH treatment stimulated IGF-1 production and this stimulated skeletal growth. In the affluent nations of Europe, cross-sectional surveys of healthy children and adolescents [18] as well as birth cohort studies of healthy children [19] find moderate-to-strong positive associations between serum IGF-1 levels and concurrent measures of height. The authors of the birth cohort study report a positive association of serum IGF-1 at age 5 and 7 years with amount of growth in height at ages 8, 9 and 10 years.

To be sure, other factors are critically important regulators of height growth, such as nutrition, infection and other diseases, psychological and emotional status, as well as genetic and epigenetic inheritance and modification [2,20,21]. Some of these growth regulators have their effect on the GH/IGH-1 axis either directly or via pathways which influence glucocorticoid and insulin physiology [22]. The important point we make here is that the positive association between variation in IGF-1 levels and height, across the range from deficiency to excess, helps to explain differences in stature within and between human populations.

In this article we test the hypothesis that differences in social status between groups of people within a population may induce variation in IGF-1 levels and, by extension, growth in height. Our hypothesis may add to understanding of why members of higher socio-economic status groups within human societies are, on average, taller than members of lower socio-economic classes [2,23,24].

## 2. Theoretical Background and Literature Review

We build our hypothesis on research into social networks, which are known to shape human behaviour and biology [25]. In their review, Christakis and Fowler summarize evidence published by their research team that interpersonal relationships influence obesity, smoking, alcohol consumption, health screening, happiness, loneliness, depression, sleep, drug use, divorce, food consumption, cooperative behaviour, influenza, sexuality and sexual orientation, and tastes in music, books, and movies. Their analyses are based on three generations of cohorts participating in The Framingham Heart Study. Residents of the town of Framingham, MA in the United States were first enrolled in 1948 and then additional cohorts were enrolled in 1978 (including many children of the first cohort), and 2002 (all children of the second cohort). One possible criticism of these findings is that the clustering of so many behaviours, psychological states, illnesses, and, even, obesity in this series of cohorts might be an artefact of the peculiarities of social life in this one American town. Another criticism is that the statistical methods used to identify social networks are inadequate [26]. Evidence against the cohort effect of one American town is quite strong and comes from studies of social networks from other geographic localities, including risk for obesity at a French high school and type-II diabetes in Iran [27]. The validity of the statistical methods used for social networks has also been addressed [28].

We apply the ideas that guide social network research to our hypothesis for a *community effect on height*. The community effect on height is a hypothetical explanation for the clustering of final adult stature within groups of people who have a high likelihood of being members of the same social network [29,30,31]. We build on the work of Hermanussen and colleagues, who proposed a community effect in height based on observations of height distributions from historical 19th and 20th century records of military conscripts. The historical conscript data are all for young men and have been used by human biologists and historians to provide a representation of male heights in a population. Aßmann and Hermanussen [29] noted that in the 19th century European people were short, for example, in 1863 the average Dutch conscript reached 165 cm, and <1% of these conscripts reached the mean body height of modern Dutch men of 184 cm. Thirty percent of the historic conscripts failed to reach 157 cm, which is less than the 1st percentile of the modern Dutch growth charts. Short stature was also noted from 19th century Switzerland and other European countries. Aßmann and Hermanussen find it peculiar that from the 19th to late 20th century the height distributions of Dutch, Swiss and other European conscripts tended to shift *in toto*, with little overlap between historic and modern height (see their Figure 1).

Using a Bayesian modelling approach and data from a longitudinal study of school children and adolescents from Zurich, Switzerland, Aßmann and Hermanussen find that in addition to well-known predictors of adult height, such as bone age and Tanner stages of puberty, there is evidence for a new parameter that they define as ‘past relative height.’ This parameter operates during the adolescent growth period to adjust the growth rate of an individual toward the average height of her/his immediate community. The authors take mathematical measures against the possibility of the spurious influence of the statistical artefact of regression to the mean. Their new parameter seems to control for individual tempo differences in growth. Aßmann and Hermanussen write, “… the smaller the adolescent is compared with past mean average height [of the community], the more the adolescent grows during puberty” ([29] p. 90). Conversely, taller than average adolescents will grow less. The net outcome is that the distribution of heights of members of a community (or a social network) will cluster toward the mean value.

**Figure 1:**
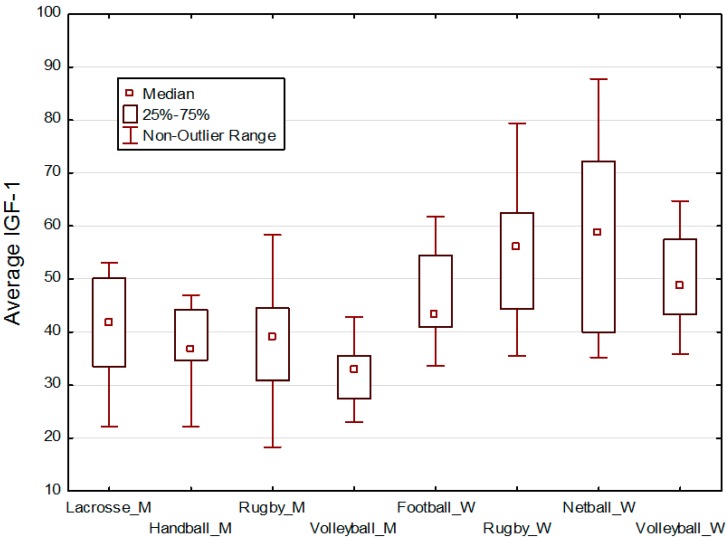
Box & whisker plots of IGF-1 values by type of sport. Pre-game and post-game IGF-1 values were averaged by addition and then division by 2. Men’s sports are designated by a ‘_M’ and women’s sports by a ‘_W’ after the name of the sport. Women in all types of sports ten to have higher IGF-1 values compared with men.

Empirical support for this community effect on height comes from analysis of Swiss conscripts measured in 1884–1891, in 1908–1910, and in 2004–2009 [30]. The authors calculated the mean height within 169 districts (political sub-divisions of Switzerland’s *cantons*) and then found that mean heights were correlated by distance between the districts (*p <* 0.01). Random network analyses suggested a direct road effect on height–closer distance by road, and not ‘as the crow flies’ direct distance, resulted in a greater correlation in height between districts. The analyses controlled statistically for income variation and for iodine deficiency (goitre prevalence) between districts, suggesting that the spatial association of body height among the Swiss conscripts is incompletely explained by wealth or health. Hermanussen and colleagues suggest, “… that people may simply be short because their friends and neighbours are short; or tall because their friends and neighbours are tall” (p. 13). This is the community effect on height, due to, perhaps, psycho-biological effects on growth and development within networks of people who directly or indirectly interact with each other because they are linked by roads.

Based on these findings it is possible to ask several questions: (1) is adult height of a community, or social network, directed toward a ‘target’, in the sense of Tanner’s [32] growth regulation of body size mammals as a processes that seeks a species-specific adult size?; (2) if so, who or what sets the target for final height?; (3) which mechanisms direct height toward the target? Hermanussen and colleagues do not discount the traditional influences of good nutrition, health, and other material privilages of higher socioeconomic status that associate with greater growth in height. They do not, however, consider these traditional influences to be sufficient to explain the *in toto* changes in height distribution over time and the height clustering within communities.

Hermanussen and colleagues suggest that the regulation of IGF-1 production, transport, and uptake at the tissue level might explain the community effect. In their view, it is likely that IGF-1 action is influenced by a variety of biological, social and emotional factors with complex interactions. The timing of the community effect to the adolescent growth period makes sense in terms of the normal age-dependent pattern of IGF-1 production. Serum IGF-1 levels are low at birth, tend to increase slowly with age, peak following puberty and during the adolescent growth spurt in height, and then decrease as growth rate slows and adult stature is achieved [12].

IGF-1 production and/or serum concentrations not only are strongly associated with skeletal growth but also with emotional status, mood, and social status. Psychosocial growth retardation, a nonorganic failure to thrive not caused by inappropriate food intake, has long been associated with neuroendocrine disturbances, especially of the GH axis [33]. Moreover, psychosocial growth retardation is preceded by psychological harassment or other types of physical or emotional stress. Children and youth with the condition are characterized with emotional depression as well as short stature and delayed puberty. In other clinical cases of short stature, an association between GH deficiency and childhood depression has long been known [34].

At present, only few studies deal with the association between social status and IGF-1. Most of the literature deals with non-human species. Even so, the GH/IGF-1 axis has an ancient evolutionary history and many of its physiological actions are conserved across species, including evolutionarily ancient pathways for tissue specific growth [35,36].

An association between social dominance, higher IGF-1levels and greater growth is also ancient and found in many Classes and Orders of the Animal Kingdom. Socially dominant Nile tilapia fish (*Oreochromis niloticus*) had higher levels of IGF-1 than subordinates [37]. Subordinates were food restricted compared with the dominants, but after controlling for the effect of food intake the subordinates still had lower IGF-1 levels and reduced growth. An experimental study with the South American pudu deer (*Pudu puda*) found that dominate males had higher IGF-1 levels during the season for antler growing and establishing territories compared to the subordinate males [38].

Social subordinance was associated with a relative suppression of IGF-1 concentrations in captive male baboons [39]. The authors report that the association was not a function of age, basal hypercortisolism of subordinate animals, differences in the quality or quantity of food consumed, basal testosterone concentrations, or genetics. It seemed, rather, that the individual differences in IGF-1 levels were a consequence, rather than a cause, of the social rank differences.

We know of only one published study of the association between social rank and IGF-1 levels in the human species. This is an analysis by Kumari and colleagues’ [40] of the 1958 British Birth Cohort. Members of the cohort were measured and interviewed at ages 42, 44 and 45 years (men, *n =* 3374; women, *n =* 3302). Social class was measured by father’s occupation at the time of the participant’s birth and also by the participant’s own occupation at age 42 years. The study team found a significant positive association in both men and women between adult IGF-1 levels and the participant’s adult social class. Women, but not men, showed a significant association between their father’s social class and their own adult IGF-1 levels. This may be because the social class of the adult women tended to be similar to their father’s social class. In contrast, many of the men in the birth cohort tended to change social class from father’s status at the time of their birth to a new status at age 42 years. It is likely, therefore, that IGF-1 levels of the men reflected current social class. The findings for women and men were statistically independent of associations between adult levels of IGF-1 and a wide range of known confounders (e.g., age, ethnicity, exercise, alcohol consumption and fatness).

## 3. Hypotheses

Our general hypothesis is that changes in social status within a well-defined social network will have an association with serum concentrations of IGF-1. Our social networks are university students involved in elite-level sporting teams. The participants in our study play at a physically and emotionally intense level of competition and their sporting activities are a major part of their university social life. We propose three specific hypotheses: (1) sport players who will win their sporting competition will have higher IGF-1 values before the game is played–this is our proxy for social dominance; (2) winners of sporting competition will increase IGF-1 levels and losers will decrease IGF-1 levels–this is our proxy for change in social dominance; (3) the IGF-1 levels of participants within a sport team will be more similar than between sport teams–this is our proxy for a social network.

## 4. Methods and Materials

### 4.1. Participants

The participants of this study were elite level sport players from undergraduate student teams. These student-athletes attended Loughborough University in the United Kingdom. Loughborough University is a world-ranked centre for competitive sport training and competition (http://loughboroughsport.com/about-us/). All of the participants in this study played on teams which competed at the highest league or championship level of the British Universities & College Sport (BUCS) organisation. Potential participants were identified and approached by student-researchers of one of the present authors (BB). These student-researchers were also athletes and able to identify high-level competitive sports players with a demanding physical training schedule and with a strong emotional commitment to success in their performance. Athletes with these characteristics are most likely to have the greatest biological and socio-emotional impact on IGF-1 levels.

We measured only student-athletes playing for a team from Loughborough University who were in competition with a team from another university. We did not include student-athletes playing for the opposing team. We did this to concentrate on the within-university social networks of the team members and not introduce analytical complications for between-university social networks.

A total of 137 participants were recruited (56 women) from 12 sport teams. Men’s sports were lacrosse, handball, rugby and volleyball. Women’s sports were football (soccer), rugby, netball and volleyball. Due to missing data a final sample of 116 participants (45 women) was available for analysis. Two phases of data collection took place, the first in 2012 and the second in 2014. The first phase yielded 36 participants (15 women) and the second phase 80 participants (41 women) with sufficient data for analysis. Participants completed a health screen questionnaire and were asked about known health issues. None had any reason for not taking part in the study. Creatine and similar supplements can influence the GH/IGF-1 axis [41,42]. None of the participants admitted to taking acute amounts of creatine, any growth hormone or any drug/protein supplements at the time of this study.

### 4.2. Variables Measured

In 2012 the variables included age, sex, type of sport, a finger prick blood spot 24 h before a sport competition and another blood spot 24 h after the competition. In 2014 the same variables were collected and also height and weight.

### 4.3. Ethical Clearance

All participants were informed about the requirements and potential risks involved with participating and gave informed written consent. The study was conducted in accordance with the Declaration of Helsinki, and the protocol was approved by the Loughborough University Ethical Advisory Committee Ethics Committee (identification code GO3-P8).

### 4.4. IGF-1 Sampling and Analysis

A finger-prick blood spot was taken from participants approximately 24 h before a competitive sporting event and then again approximately 24 h after the event. Rates of change for IGF-1 are relatively slow. One clinical study found increased levels of serum IGF-1 occurred 4–6 h after stimulation by administration of GH, and that IGF-2 and serum IGFBP-3 (the major binding protein for the IGFs) steadily increased for about 36 h, when the blood sampling ended. [43]. By collecting blood samples approximately 24 h before and after a competition we expected to observe near maximal change in in the GH/IGF-1 axis.

All data collection was carried out by five students as part of their Final Year Honours Research Project. All students were trained in anthropometry by BB and trained to take the blood spots by a licensed phlebotomist. Data were collected at laboratory at Loughborough University. Height was measured with a wall-mounted Harpenden anthropometer, weight with a Tanita balance, and blood spots were saved onto Whatmann Protein Saver™ 903™ Cards. All sporting competitions took place on Wednesday afternoons at approximately the same time of day. Blood spot collection took place weekly on Tuesdays and Thursday between 2–4 pm, approximately 24 h before or after a competition.

Measuring IGF-1 from dried blood spots on filter paper is both practical and reliable [44]. The analysis of the blood spot samples was carried out by one well qualified technician at the IGF/Peptide Hormone Laboratory at the Children's Hospital of Giessen (Universitätskinderklinik Giessen), Germany. Samples for the IGF-1 RIA were prepared by punching out two discs per blood spot from the Protein Saver Cards which corresponds to 6.25 µL blood. The two punches were extracted in 200 µL 0.9% (w/v) NaCl solution at 4 °C overnight and were then further diluted with 200 µL twofold concentrated RIA assay buffer. Duplicates of 100 µL filter paper extract were assayed by IGFBP-blocked RIA following the method described by Blum and Breier [45]. All samples were extracted and measured in one single assay to avoid inter-assay variation. The intra-assay coefficient of variation of this RIA was 1.6% and sensitivity with the applied dilution scheme was 0.48 µg/L. IGF-1 values derived from dried blood are reported here; conversion to serum IGF-1 values requires multiplication by 4.2.

## 5. Results

Descriptive statistics for age, height, weight, pre-game and post-game IGF-1 are given in Table 1 for men and Table 2 for women. Box plots of averaged pre-game and post-game IGF-1 values by type of sport are presented in Figure 1.

**Table 1:** Descriptive statistics for men.

	Age, Years	Height, cm	Weight, kg	Pre-IGF-1 (µg/L)	Post-IGF-1 (µg/L)
MEN (*n =* 71)					
Mean	21.3	182.2	88.1	37.8	38.1
SD	1.5	6.4	10.6	9.9	10.7
Min	19	168.6	62.5	20.2	16.5
Max	25	195.0	109.0	66.6	60.0

**Table 2:** Descriptive statistics for women.

	Age, Years	Height, cm	Weight, kg	Pre-IGF-1 (µg/L)	Post-IGF-1 (µg/L)
WOMEN (*n =* 45)					
Mean	20.1	171.0	69.2	53.0	54.3
SD	1.3	7.2	8.9	14.2	15.7
Min	18	157.0	56.8	27.5	33.0
Max	23	191.0	95.0	88.5	87.0

Multiple regression was performed to see if pre-game IGF-1 levels (the dependent variable) were associated with Age, Sex, Height, Weight or type of Sport (independent variables). A significance level of *p* ≤ 0.05 was used for this and all other analyses. Results are given in Table 3. Age and Sex are significantly associated with pre-game IGF-1. Younger participants and women have higher IGF-1 values than older participants and men.

**Table 3:** Wilkes multivariate tests of significance from multiple regression.

	Value	F; DFs	*p*
Intercept	0.89	2.95, 2, 50	0.06
Age, years	0.78	6.89	0.002
SEX	0.75	8.18	0.0008
Height cm	0.96	0.95	0.39
Weight kg	0.99	0.37	0.69
SPORT	0.91	2.53	0.09

The age effect is expected in our 18–25 year old participants, as younger adolescents and adults in this age range tend to have higher IGF-1 levels than older individuals. The women participants are, on average, younger than the men, and this accounts for some of the Sex effect. It is possible that selection bias also is a factor. Elite women athletes are a highly selected group and may have high testosterone production, which leads to higher GH and IGF-1 levels [22,39,41].

When the multiple regression analysis was performed by sex, then Age was significant for the men, but not significant for the women. There was no association between Age and type of Sport, so we were able to combine IGF-1 data for all sports. Due to the significant Sex effect, all tests of our hypotheses were conducted separately for men and women.

Our first specific hypothesis is that sport players who will win their sporting competition will have higher IGF-1 values before the game is played. We found possible support for this hypothesis. Comparing mean (standard deviation, sample size) pre-game IGF-1 levels finds that all winners = 46.4 µg/L (13.8, 45) and all losers = 41.6 µg/L (14.4, 43). A one-sided *t*-test is not quite statistically significant at *p =* 0.057. A one-sided *t*-test was chosen as we hypothesised *a priori* that winners would have higher IGF-1 values than losers. Comparisons of post-game IGF-1 values for winners *vs.* losers found no differences.

Another way to test for the effect of winning *vs.* losing is to compare the mean values of the combined pre-game and post-game IGF-1 for all winners against all losers. In this statistical test, the winners had a combined mean IGF-1 value of 46.2 µg/L (13.8, 55) and the losers had a combined mean value of 41.6 µg/L (12.4, 46). A one-sided *t*-test is statistically significant at *p =* 0.042. This finding suggests that the eventual winners had higher IGF-1 values both 24 h before and 24 h after the sporting competition. Testing by sex found similar trends, but a smaller absolute difference between winners and losers and no significance.

Our second hypothesis is that changes in social status based on winning or losing important sporting competitions will be associated with changes in IGF-1 serum concentration in the players. There was no support for this hypothesis. Using a dependent *t*-test, we found no significant difference between pre-game and post-game mean values for IGF-1 for men, for women, or for both sexes combined. For individual participants there was no pattern of increase or decrease in IGF-1 values from pre- to post-game related to winning or losing.

Our third hypothesis is that IGF-1 levels of participants within a sport team will be more similar than between sport teams, that is, within a social network of players who know each other well. There was no support for this hypothesis. As may be seen in Figure 1, for each sex the range of IGF-1 values by sport is large relative to the differences in mean IGF-1 values.

## 6. Discussion

The relationship between insulin-like growth factor-1 (IGF-1), assessed via finger-prick dried blood spot, and elite level sport competition outcome was analysed for a sample of 116 undergraduate men and women attending a British university. There was a statistically significant difference between the mean values of the combined pre- and post-game IGF-1 for all winners *vs.* all losers the sport competitions. Winners, as a group, had a 4.6 µg/L higher average pre-game and post-game IGF-1 levels than losers. We did not predict this specific finding, but it is generally supportive of our hypothesis 1 which we proposed this type of difference as our proxy for social dominance. The difference in mean values amounts to about 11% greater serum IGF-1 for the winners. The biological impact of this difference for the participants of this study is not known, but the existing research reports strong positive associations between greater IGF-1 and greater body skeletal growth, physical performance, emotional status and physical and mental energy [12,46,47]. Other research finds that a 1.0 µg/L increase in serum IGF-1 is associated with higher cognitive performance in children [48]. Higher cognitive performance is a key to both sporting success and social dominance [49,50,51].

We found no evidence that winners increased, or decreased, in IGF-1 levels over losers (hypothesis 2). It seems that winners already have, on average, higher IGF-1 values the day before the game and that this difference is maintained for at least 48 h. We found no evidence that members of the same team were more similar in IGF-1 levels than they were to players from other teams (hypothesis 3). A possible explanation for the hypothesis 3 results is that despite being on different teams, all of these elite student-athletes from the same university were actually part of the same social network. Future studies of this type should measure the degree of interaction in terms of training and social activities of the participants.

These findings provide limited support toward the community effect in height hypothesis. The support comes from the possibility that the higher total pre-game and post-game IGF-1 of the winners was due to their persistent social dominance over the losers.

A hierarchy of social dominance results when members of a social group vary in their ability to compete for resources or attention. Social dominance is usually measured via contests between two or more individuals, with winners ranked as dominant and losers ranked as subordinate [52,53]. Ethological research with non-human and human species has revised older notions that dominance is only achieved via coercive or aggressive behaviours. Dominant individuals or social groups may be prosocial as well as coercive toward subordinates. The very large and diverse literature on the endocrinology of dominance is mostly focused on the hypothalamic-pituitary-adrenal (HPA) axis and its hormonal end-products, such as cortisol [54,55]. In the Introduction to this article we reviewed the limited literature related to social dominance and IGF-1. It is important to note that there are many neuroendocrine pathways which connect the HPA axis with the GH/IGF-1 axis [12,22,56,57], but we did not measure any direct HPA activity, nor did we measure cortisol.

Sporting contests between elite athletes is a justifiable model system to study social dominance and its associations with IGF-1. Elite level sport requires a rare combination of talent, hard work and the right psychological profile, often a mixture of confidence, anxiety, and motivation [51]. Research comparing elite level team handball players with lower level players finds that the elites are, on average, significantly different in terms of being physically larger in both height and muscle mass, faster and more agile, possess superior skills in game performance and tactics, are more emotionally resilient, more team oriented, come from higher socioeconomic status families and have greater ego motivational orientation [50].

The associations of greater height, socioeconomic status and ego motivation in sporting success are also predicted components of the community effects in height hypothesis. It is, of course, difficult to disentangle cause and effect in the bio-psychological profile of sport players’ characteristics. Similarly, it is not possible to completely segregate causes and effects in the regulation of growth in height.

Simple cause and effect relationships may not be the appropriate perspective to adopt in sport competition or in human height growth. Both sport results and growth in height are the outcome of many biological, nutritional, social, economic, political and psychological effects and interactions [2,5,31,49,51,58,59,60]. An information- and systems-based network approach to understanding the regulation of growth in height seems more appropriate, as has been adopted in recent research on the GH/IGF axis [57], evolutionary biology and genomics [61] and in the growth and maturation of commercially important livestock [62].

### Limitations

Interpretation of the findings of our study is also limited by other factors. These include: (1) a modest sample size; (2) no assessment of the amount or intensity of training in the days prior to the competition—which may increase IGF-1 until a fatigue threshold is reached and then decrease IGF-1; and (3) no testing for ‘doping’ with GH and other banned supplements. We did ask participants to list all performance supplements taken and a few admitted to creatine, which can enhance IGF-1 production [63]. The senior author asked the students who collected the data for this study about creatine usage. These students, all of whom were themselves athletes, disclosed that they and all their team-mates used creatine and protein supplements. This included both the men and the women. If this is true, then the creatine effect on IFG-1 levels was virtually equalized for all participants. Finally, we did not measure dietary intake, including alcohol, before or after the competition. It is known that the response of IGF-1 to overeating is minimal and that the response to complete fasting is relatively slow, with a decrease of about 5% after 24 h and 30% after 72 h [12]. It is unlikely that our participants fasted at any time. Chronic alcohol abuse is known to decrease IGF-1 production due to ethanol-induced liver injury [64], but again it is unlikely that our participants were liver injured or chronic alcohol abusers.

## 7. Conclusions

Our findings offer some support for further investigation of the community effect in height hypothesis. In essence, this hypothesis posits that there are influences on the attainment of final height which arise from the bio-social-psychological proximity of members within a social network. A next step in testing this hypothesis is validation of the findings by repeating the study with larger samples. Such validation may warrant new analyses of longitudinal studies of IGF-1 levels of children and youth according to their social networks.

Network analysis may be able to help move the study of human growth away from an unproductive ‘genes v environment’ notion that is still popular with non-scientists and also make an advance on the simple ‘genes x environment’ interaction model that became out-dated with the empirical observation of epigenetic mechanisms regulating development of the phenotype [20,36,57,65,66,67]. A network approach centred on the GH/IGF-1 axis should be especially productive because of the observations of Blum and colleagues ([12], p. 159) that:

GH secretion itself is regulated by endogenous signals coming from the central nervous system (e.g., the increase in GH during puberty) or by chronic psychosocial or physical distress (e.g., psychosocial growth retardation or extreme training load in high-performance athletes). In addition, the immune system may be involved and, when activated, it causes suppression of IGF-1… In the event that one of these major regulators is missing (GH or nutrients) or activated (immune system), there is resistance against the other factors with respect to IGF-1 production. That is, IGF-1 transmits integrated information at the cellular level on the nutritional status, the GH secretory status, and the immune status of the organism. Generally speaking, IGF-1 provides information to the cells on the well-being of the organism. Thus, the rate of cellular activities such as proliferation, differentiation, or the synthesis of cell-specific products is adapted to the situation. “*Evidently, this kind of signal is of the utmost importance to the growing organism*” (emphasis added).

As succinctly stated in the above quote, many types of biological, social, economic, political, and emotional networks influence human growth. There are at least three types of networks specifically mentioned or implied within the present analysis: (1) Social and (2) Physical (height), which we describe in this article; and (3) Genomic/Evolutionary, which is beyond the scope of the present analysis but is treated elsewhere [36].

The GH/IGF-1 axis serves as a transducer of these multiple influences into a coherent signal which allows the growing human to adjust and adapt to local ecological conditions. This serves to emphasise why human growth in height serves as a sensitive indicator of well-being by researchers in environmental epidemiology and public health. Further testing of the community effect on height hypothesis should continue to focus on the action of the GH/IGF-1 axis within an information- and systems-based approach.
